# Reply to “Intrinsic protein disorder uncouples affinity from binding specificity”

**DOI:** 10.1002/pro.4601

**Published:** 2023-04-01

**Authors:** Tamás Lázár, Agnes Tantos, Peter Tompa, Eva Schad

**Affiliations:** ^1^ VIB‐VUB Center for Structural Biology, Flanders Institute for Biotechnology (VIB) Brussels Belgium; ^2^ Structural Biology Brussels Vrije Universiteit Brussel Brussels Belgium; ^3^ Institute of Enzymology, Research Centre for Natural Sciences Budapest Hungary

First of all, we would like to acknowledge that it is our fault that we failed to cite your work—we simply missed it, which can only be explained by the long time passing between the conception and completion of our research article (*Full‐length paper*).

With reference to the arguments on free energy of binding, the comparison of binding thermodynamics between ORD‐ORD and ORD‐IDP complexes has been carried out a number of times before, even before your study published in 2015, for example, by Dogan et al. (2014), Huang and Liu (2009), Liu and Huang (2014), Vacic et al. (2007), and Zhou (2012). We had no intention in presenting these distributions as novel results, simply as a basic descriptor of our datasets before presenting the seven other related chapters.

**FIGURE 1:**
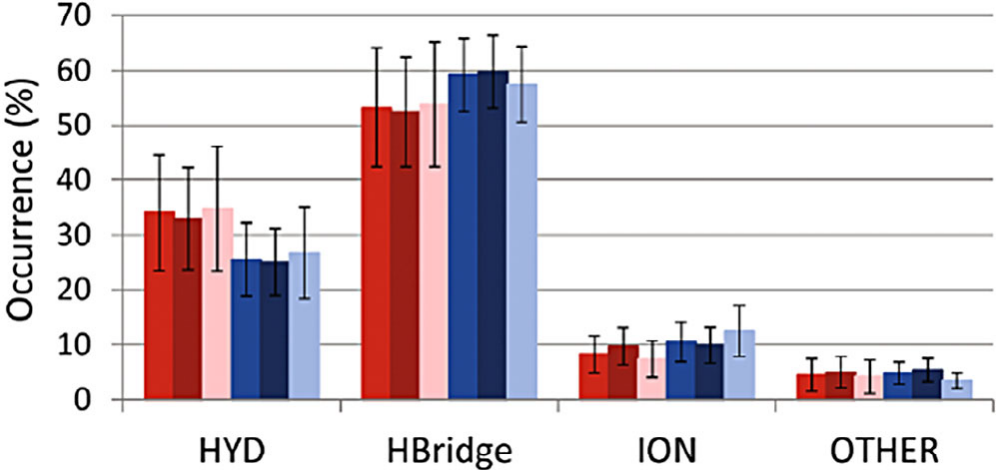
Interaction types of ID and globular complexes. (a) Percental distribution of interaction types (HYD: hydrophobic; HBridge: hydrogen bond; ION: ionic; OTHER: disulfide bridges, aromatic–aromatic, aromatic–sulfur, and cation–pi interactions) in strong and weak complexes (red: all ID complexes; dark red: strong; rose: weak ID complexes; blue: all globular complexes; dark blue: strong; light blue: weak globular complexes). Error bars represent mean ± SD. Original figure: fig. 5a in Lazar et al. (2022).

Second, as our work was completed later, we could collect many more examples, and could exclusively focus on cases for which high‐resolution structural data were available (in Teilum et al. (2015); there are 52 such ORD‐ORD cases and 41 ORD‐IDP cases, whereas in our study (Lazar et al., 2022), we have analyzed 144 ORD‐ORD and 259 ORD‐IDP complexes, respectively). As a result, our statistics shows some deviations from that reached in Teilum et al. (2015), in the sense that IDPs have significantly more hydrophobic residues in their interfaces (Figure 1), as also suggested earlier (Fuxreiter et al., 2007; Meszaros et al., 2007). This is somewhat surprising as IDPs in general have much less hydrophobic residues than globular proteins, but apparently, most of these serve protein–protein interactions. Desolvation accompanying hydrophobic binding may provide a favorable entropic contribution, tempering the conclusion that the large loss of conformational entropy associated with binding dominates ORD‐IDP binding energy, making it in general weaker than ORD‐ORD.

With regard to the issue of specificity, we find that definitions surrounding specificity are way more diverse than a single metric that the whole community can basically agree on. As outlined below, we must distinguish “defining” specificity theoretically and “understanding” some flavors of specificity based on evolutionary or chemical features, from “measuring” it in absolute terms, which we think cannot be done. That is, we accept that specificity can be defined.

1. Thermodynamically (as favorable balance of binding equilibria
  - of functional vs. all nonfunctional competing interactors; or
  - of true binding site versus all the other binding patches on the same protein; or
  - of wild‐type interface vs. mutated interface of the protein);
2. Chemically (interactions that show a high level of chemical complementarity); and also
3. Evolutionarily (conserved half‐interfaces of complexes that co‐evolved and may even exhibit detectable sequence covariation).

The problem, as we see it, is that these are theoretical definitions, which can be quantified for a given A:B protein pair but in practice cannot be assessed beyond single proteins of interest, for example, on the whole proteome (which should be done for properly quantifying specificity factor a (Teilum et al., 2021) for a thermodynamics‐based definition). As we most importantly do not know the parameters of all competing interactions, we are not in the position to tell the thermodynamic specificity of a given interaction. A closely related problem is that in principle, the authors of both papers (Lazar et al., 2022; Teilum et al., 2015) only studied “specific” interactions, without a control dataset of false positive cases. Furthermore, it is well‐accepted that there are still many more true positive interactions in the proteome that still await for discovery, and this introduces another complication left undiscussed. These challenges are the very reasons we suggest considering orthogonal measures for thermodynamic specificity, such as (i) evolutionary conservation, (ii) patterning/information content of the interface, and (iii) functional similarity of interaction partners, all of which arguably put specificity in a more biological context without the need of having to assess specificity for nonbinding or nonspecific partners for quantifying it.

As a result, we hold our conclusion that specificity can be uncoupled from affinity, not in a sense that a weaker interaction would win over a stronger one (although in the cell it could), rather than a weak interaction can be as specific as a strong one, against the background of even weaker competitors. In this sense, our conclusion does not fall far from that reached by Teilum et al. (2021), that is: “…low affinity and high specificity as a generic property for IDPs. There may in fact be some truth to it. Although we have seen many IDPs binding with high affinity, even in the low or sub nM range, the higher degree of multispecificity and potentially lower specificity factors, combined with high adaptability, may indeed require low affinity for generating high specificity (competing interactions might be even weaker).” Or “even highly nonspecific, low affinity and accidental interactions can have biological relevance. This means that the degree of specificity, the magnitude of the *K*_d_s and biological relevance are not necessarily correlated” (Teilum et al., 2021).

Sincerely,

Tamas Lazar

Agnes Tantos

Peter Tompa

Eva Schad
